# Self-assembly of organogels via new luminol imide derivatives: diverse nanostructures and substituent chain effect

**DOI:** 10.1186/1556-276X-8-278

**Published:** 2013-06-10

**Authors:** Tifeng Jiao, Qinqin Huang, Qingrui Zhang, Debao Xiao, Jingxin Zhou, Faming Gao

**Affiliations:** 1 Hebei Key Laboratory of Applied Chemistry, School of Environmental and Chemical Engineering, Yanshan University, Qinhuangdao, 066004, China; 2 State Key Laboratory of Solid Lubrication, Lanzhou Institute of Chemical Physics, Chinese Academy of Sciences, Lanzhou, 730000, China

**Keywords:** Nanostructure, Self-assembly, Organogel, Luminol derivative, Substituent group effect

## Abstract

Luminol is considered as an efficient sycpstem in electrochemiluminescence (ECL) measurements for the detection of hydrogen peroxide. In this paper, new luminol imide derivatives with different alkyl substituent chains were designed and synthesized. Their gelation behaviors in 26 solvents were tested as novel low molecular mass organic gelators. It was shown that the length and number of alkyl substituent chains linked to a benzene ring in gelators played a crucial role in the gelation behavior of all compounds in various organic solvents. Longer alkyl chains in molecular skeletons in present gelators are favorable for the gelation of organic solvents. Scanning electron microscope and atomic force microscope observations revealed that the gelator molecules self-assemble into different micro/nanoscale aggregates from a dot, flower, belt, rod, and lamella to wrinkle with change of solvents. Spectral studies indicated that there existed different H-bond formations and hydrophobic forces, depending on the alkyl substituent chains in molecular skeletons. The present work may give some insight to the design and characteristic of new versatile soft materials and potential ECL biosensors with special molecular structures.

## Background

The self-assembly of small functional molecules into supramolecular structures is a powerful approach toward the development of new nanoscale materials and devices [1-7]. As a novel class of self-assembled materials, low weight molecular organic gelator (LMOG) gels organized in regular nanoarchitectures through specific noncovalent interactions including hydrogen bonds, hydrophobic interaction, π-π interactions, and van der Waals forces have recently received considerable attention [8-13]. Up to now, LMOGs have become one of the hot areas in soft matter research due to their scientific values and many potential applications in wide fields, including nanomaterial templates, biosensors, controlled drug release, medical implants, and so on [14-19]. The noncovalent nature of the 3D networks within the supramolecular gels promises accessibility for designing and constructing sensors, actuators, and other molecular devices [20-23].

In addition, in the recent several decades, luminol is considered as an efficient system in chemiluminescence and electrochemiluminescence (ECL) measurements for the detection of hydrogen peroxide [24-27]. In the previous work, we reported the design and synthesis of functional luminol derivatives with different substituted groups and investigated the interfacial assembly of these compounds with different methods [28,29]. Therein, their potential for ECL measurement has been demonstrated first. Meanwhile, their interfacial behavior and the morphologies of pure or mixed monolayers used to develop the biomimetic membrane were investigated [30]. The introduction of different substituted groups into those functional compounds can lead to new conjugated structures, and new properties are expected. Furthermore, in our reported work, the gelation properties of some cholesterol imide derivatives consisting of cholesteryl units and photoresponsive azobenzene substituent groups have been investigated [31]. Therein, we found that a subtle change in the headgroup of the azobenzene segment can produce a dramatic change in the gelation behavior of two compounds with/without methyl substituent groups described therein. In addition, the gelation properties of bolaform and trigonal cholesteryl derivatives with different aromatic spacers have been characterized [32]. Therein, we have investigated the spacer effect on the microstructures of such organogels and found that various kinds of hydrogen bond interactions among the molecules play an important role in the formation of gels.

In this study, we have designed and synthesized new luminol imide derivatives with different alkyl substituent chains. In all compounds, the different alkyl chains were symmetrically attached to a benzene ring to form single/three substituent states, with the luminol segment as substituent headgroups. We have found that most compounds could form different organogels in various organic solvents. Characterization of the organogels by scanning electron microscopy (SEM) and atomic force microscopy (AFM) revealed different structures of the aggregates in the gels. We have investigated the effect of the length and number of alkyl substituent chains in gelators on the microstructures of such organogels in detail and found different kinds of hydrogen bond interactions between amide groups.

## Methods

### Materials

**Figure 1:**
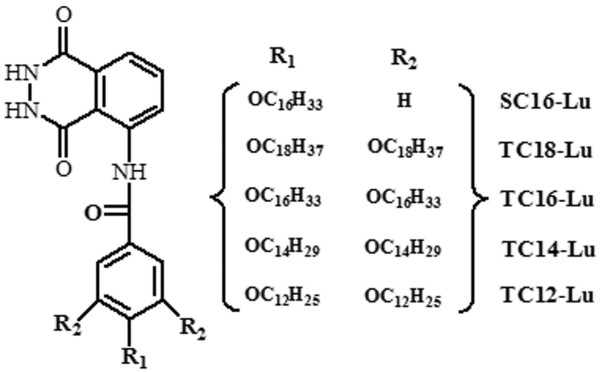
Molecular structures and abbreviations of these luminol imide derivatives.

The starting materials, luminol (3-aminophthalhydrazide), methyl 3,4,5-trihydroxybenzoate, 1-bromooctadecane, 1-bromohexadecane, 1-bromotetradecane, and 1-bromododecane, were purchased from Alfa Aesar Chemicals (Ward Hill, MA, USA) or TCI Shanghai Chemicals (Shanghai, China). Other used reagents were all for analysis purity from Alfa Aesar Chemicals or Aldrich Chemicals (Sigma-Aldrich Corporation, St. Louis, MO, USA), respectively. The solvents were obtained from Beijing Chemicals (Beijing, China) and were distilled before use. Deionized water was used in all cases. 4-Alkyloxy-benzoic acid and 3,4,5-tris(alkyloxy)benzoic acid with different substituent chains were synthesized in our laboratory according to the previous report [33] and confirmed by ^1^H nuclear magnetic resonance (NMR). Then, these luminol imide derivatives were prepared using similar methods [34,35]. Simply speaking, different benzoic acid chlorides were synthesized by heating an acid compound solution in sulfoxide chloride and dimethylformamide (DMF) (V_sulfoxide chloride_/V_DMF_ = 10:1) for about 10 h at 70°C. Then, the prepared benzoic acid chlorides reacted with luminol in dried DMF in the presence of pyridine for 3 to 4 days by using an ice bath. After that, the mixtures were washed with pure water, filtered, and dried in vacuum. The residues were purified by recrystallization in ethanol solution as yellow solids. These new products and their abbreviations are shown in Figure 1, which were confirmed by ^1^H NMR and elemental analysis. Their syntheses will be reported elsewhere on due course.

### Gelation test

A weighted amount of gelator and a measured volume of a selected pure organic solvent were placed into a sealed glass bottle, and the solution was heated in a water bath until the solid was dissolved. Then, the solution was cooled to room temperature in air, and the test bottle was inversed to see if a gel was formed. When the gelator formed a gel by immobilizing the solvent at this stage, it was denoted as ‘G’. For the systems in which only the solution remained until the end of the tests, they were referred to as solution (S). The system in which the potential gelator could not be dissolved even at the boiling point of the solvent was designated as an insoluble system (I). Critical gelation concentration refers to the minimum concentration of the gelator for gel formation.

### Characterization techniques

Firstly, these as-formed xerogels under the critical gelation concentration were prepared by a vacuum pump for 12 to 24 h. The dried samples thus obtained were attached to mica, copper foil, glass, and CaF_2_ slice for morphological and spectral investigation, respectively. Before SEM measurement, the samples were coated on a copper foil fixed by a conductive adhesive tape and shielded by gold. SEM pictures of the xerogel were taken on a Hitachi S-4800 field emission scanning electron microscope (Hitachi, Ltd., Tokyo, Japan) with an accelerating voltage of 5 to 15 kV. AFM images were recorded using a Nanoscope VIII Multimode scanning probe microscope (Veeco Instruments, Plainview, NY, USA) with silicon cantilever probes. All AFM images were shown in the height mode without any image processing except flattening. Transmission Fourier transform infrared (FT-IR) spectra of the xerogel were obtained using a Nicolet iS/10 FT-IR spectrophotometer from Thermo Fisher Scientific Inc. (Waltham, MA, USA) by an average of 32 scans and at a resolution of 4 cm^−1^. The X-ray diffraction (XRD) measurement was conducted using a Rigaku D/max 2550PC diffractometer (Rigaku Inc., Tokyo, Japan). The XRD pattern was obtained using CuKα radiation with an incident wavelength of 0.1542 nm under a voltage of 40 kV and a current of 200 mA. The scan rate was 0.5°/min. ^1^H NMR spectra were obtained on a Bruker ARX-400 (Bruker, Inc., Fällanden, Switzerland) NMR spectrometer in CDCl_3_ with TMS as an internal standard. The elemental analysis was carried out with the Flash EA Carlo-Erba-1106 Thermo-Quest (Carlo Erba, Milan, Italy).

## Results and discussion

**Figure 2:**
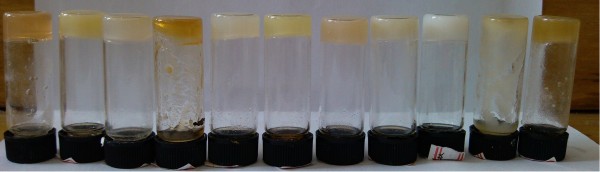
**Photographs of organogels of TC18-Lu in various solvents.** Isopropanol, cyclopentanol, *n*-butanol, DMF, aniline, petroleum ether, *n*-pentanol, nitrobenzene, ethanol, 1,4-dioxane, and cyclopentanone (from left to right).

The gelation performances of all luminol imide derivatives in 26 solvents are listed in Table 1. Examination of the table reveals that most compounds are efficient gelators, except that TC12-Lu cannot gel any present solvent. Firstly, SC16-Lu with single alkyl substituent chains in the molecular skeleton can gel in ethanolamine and DMSO. As for four imide compounds with three alkyl substituent chains in the molecular skeleton, obvious differences were obtained. TC18-Lu and TC16-Lu can gel in 11 or 12 solvents, respectively. For the cases of TC14-Lu and TC12-Lu with shorter alkyl substituent chains in molecular skeletons, the numbers of formed organogels changed to 4 and 0, respectively. The photograph of organogels of TC18-Lu in various solvents was shown in Figure 2. The data shown in Table 1 indicated that the length and number of alkyl substituent chains had a profound effect upon the gelation abilities of these studied imide compounds. It seemed that longer alkyl chains in molecular skeletons in present gelators are favorable for the intermolecular stacking and subsequent gelation of organic solvents, which was similar to the previous relative reports [36,37]. In addition, it is interesting to note that three compounds from TC18-Lu to TC14-Lu can form organogels in DMF, respectively, which can be due to the special intermolecular forces between imide compounds and solvents. The reasons for the strengthening of the gelation behaviors for TC18-Lu and TC16-Lu can be assigned to the change of hydrophobic force and the spatial conformation of the gelators due to longer alkyl substituent chains in molecular skeletons, which may increase the ability of the gelator molecules to self-assemble into ordered structures, a necessity for forming organized network structures.

**Table 1:** Gelation behaviors of luminol imide derivatives at room temperature

**Solvents**	**SC16-Lu**	**TC18-Lu**	**TC16-Lu**	**TC14-Lu**	**TC12-Lu**
Acetone	I	I	G (1.5)	I	PS
Aniline	S	G (2.0)	G (2.0)	G (1.5)	PS
Toluene	PS	PS	I	PS	PS
Pyridine	S	S	G (2.0)	S	S
Isopropanol	PS	G (2.5)	G (2.0)	PS	PS
Cyclopentanone	PS	G (2.0)	G (1.5)	PS	PS
Cyclohexanone	PS	PS	G (2.0)	PS	PS
Nitrobenzene	S	G (2.0)	G (2.0)	G (2.0)	PS
*n*-Butanol	PS	G (2.5)	G (2.0)	PS	PS
Ethanolamine	G (2.0)	PS	I	S	PS
*n*-Butyl acrylate	PS	PS	S	PS	PS
1,4-Dioxane	PS	G (2.5)	G (2.0)	S	PS
Petroleum ether	S	G (2.0)	S	S	PS
Ethyl acetate	PS	PS	S	PS	PS
Dichloromethane	PS	S	S	S	S
THF	I	PS	S	PS	PS
DMF	PS	G (2.0)	G (1.5)	G (1.5)	S
DMSO	G (2.5)	PS	I	G (2.0)	PS
Ethanol	PS	G (2.0)	G (2.0)	PS	PS
Benzene	PS	PS	I	S	PS
Tetrachloromethane	PS	PS	PS	S	S
Acetonitrile	PS	PS	PS	PS	PS
Methanol	PS	PS	S	PS	PS
*n*-Pentanol	PS	G (2.5)	G (2.0)	PS	PS
Cyclopentanol	PS	G (2.0)	S	PS	PS
Formaldehyde (aq.)	PS	PS	PS	PS	PS

*DMF* dimethylformamide, *THF* tetrahydrofuran, *DMSO* dimethyl sulfoxide, *S* solution, *PS*, partially soluble, *G* gel, *I* insoluble. For gels, the critical gelation concentrations at room temperature are shown in parentheses (% *w*/*v*).

**Figure 3:**
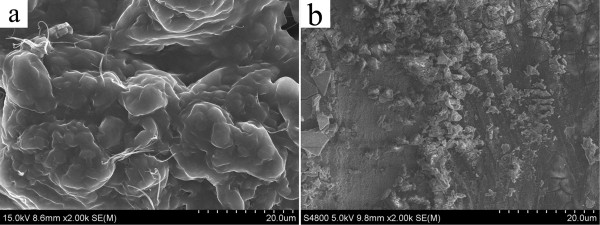
**SEM images of xerogels (SC16-Lu gels).** (**a**) Ethanolamine and (**b**) DMSO.

**Figure 4:**
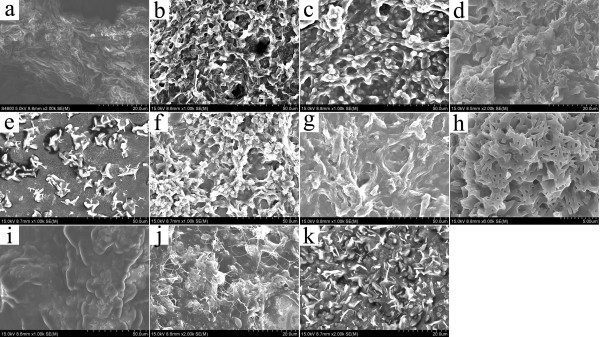
**SEM images of xerogels (TC18-Lu gels).** (**a**) Aniline, (**b**) isopropanol, (**c**) cyclopentanone, (**d**) nitrobenzene, (**e**) *n*-butanol, (**f**) 1,4-dioxane, (**g**) petroleum ether, (**h**) DMF, (**i**) ethanol, (**j**) *n*-pentanol, and (**k**) cyclopentanol.

**Figure 5:**
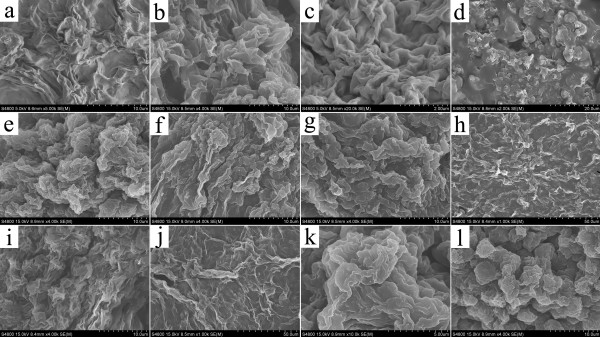
**SEM images of xerogels (TC16-Lu gels).** (**a**) Acetone, (**b**) aniline, (**c**) pyridine, (**d**) isopropanol, (**e**) cyclopentanone, (**f**) cyclohexanone, (**g**) nitrobenzene, (**h**) *n*-butanol, (**i**) 1,4-dioxane, (**j**) DMF, (**k**) ethanol, and (**l**) *n*-pentanol.

**Figure 6:**
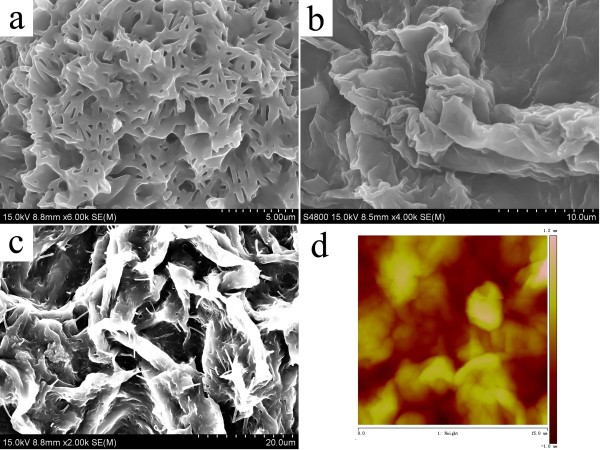
**SEM and AFM images of xerogels.** (**a**) TC18-Lu, (**b**,**d**) TC16-Lu, and (**c**) TC14-Lu in DMF gels.

In order to investigate the prepared nanostructures of various organogels, the typical nanostructures of the xerogels were studied by SEM and AFM techniques. From the images in Figure 3, it was easily observed that the SC16-Lu xerogel from ethanolamine showed large wrinkle-like aggregates in the micrometer scale, while blocks with a dot-like morphology appeared in DMSO. In addition, as seen in Figure 4, the SEM images of xerogels from TC18-Lu gels showed diverse micro-/nanomorphologies, such as dot, flower, belt, rod, lamella, and wrinkle. From Figure 5, the SEM images of xerogels from TC16-Lu gels in 12 solvents showed that the self-assembled nanoaggregates tended to stack and overlap each other to form larger multilevel aggregations. Furthermore, the morphologies of xerogels from TC18-Lu, TC16-Lu, and TC14-Lu in DMF were compared, as shown in Figure 6. With the length decrement of alkyl substituent chains in molecular skeletons, flower, lamella, and big slide with subsequently increased sizes were observed, respectively. From the AFM image of TC16-Lu in DMF, as seen in Figure 6d, it is interesting to note that these big lamella aggregates were composed of smaller domains by stacking of the present imide derivatives. The morphologies of the aggregates shown in the SEM and AFM images may be rationalized by considering a commonly accepted idea that highly directional intermolecular interactions, such as hydrogen bonding or hydrophobic force interactions, favor formation of belt or fiber structures [38-41]. The changes of morphologies between molecules with different alkyl substituent chains can be mainly attributed to the different strengths of the intermolecular hydrophobic force between alkyl substituent chains, which have played an important role in regulating the intermolecular orderly staking and formation of special aggregates.

**Figure 7:**
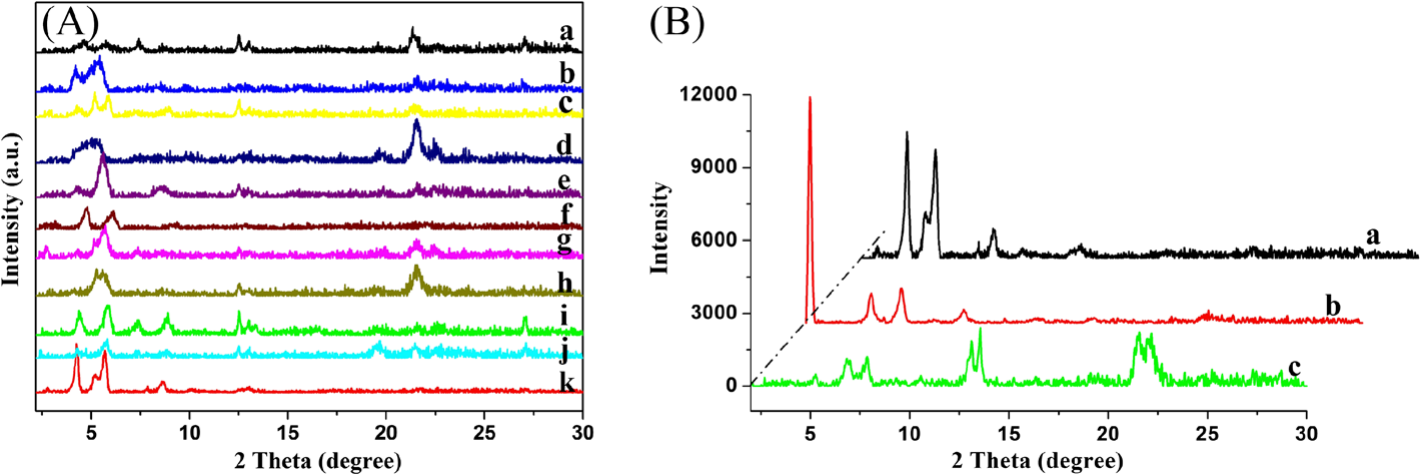
**X-ray diffraction patterns of xerogels.** (**A**) TC18-Lu (a, isopropanol; b, 1,4-dioxane; c, cyclopentanol; d, cyclopentanone; e, *n*-butanol; f, ethanol; g, *n*-pentanol; h, nitrobenzene; i, petroleum ether; j, aniline; and k, DMF). (**B**) Xerogels in DMF (a, TC18-Lu; b, TC16-Lu; and c, TC14-Lu).

In addition, in order to further investigate the orderly assembly of xerogel nanostructures, the XRD patterns of all compound xerogels from gels were measured. Firstly, TC18-Lu was taken as an example, as shown in Figure 7A. The typical curve for the TC18-Lu xerogel from petroleum ether shows main peaks in the angle region (2*θ* values, 4.42°, 5.86°, 7.36°, 8.86°, 12.52°, and 21.58°) corresponding to *d* values of 2.00, 1.51, 1.20, 1.00, 0.71, and 0.41 nm, respectively. Other curves have a little difference from the data above. The change of corresponding *d* values suggested different stacking units with various nanostructures of the aggregates in the gels [42]. In addition, the XRD data of xerogels of TC18-Lu, TC16-Lu, and TC14-Lu in DMF were compared, as shown in Figure 7B. The curves of TC18-Lu and TC14-Lu showed a similar shape with the minimum peaks at 4.26° and 5.24°, respectively. The corresponding *d* values were 2.08 and 1.69 nm, respectively. As for the curve of TC16-Lu in DMF, additional strong peaks appeared at 2.2°, with a corresponding *d* value of 4.02 nm. The value is near double of other numbers, suggesting a special stacking mode with two-molecular length. The present results described above demonstrated again that the alkyl substituent chains had a great effect on the assembly modes of these imide compounds.

**Figure 8:**
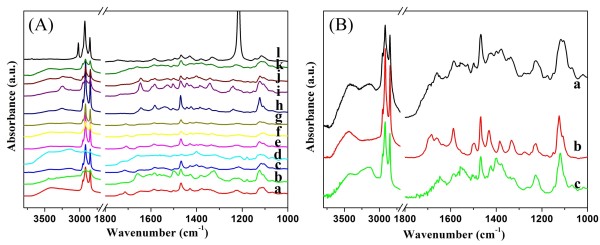
**FT-IR spectra of xerogels.** (**A**) TC18-Lu (a, isopropanol; b, 1,4-dioxane; c, cyclopentanol; d, cyclopentanone; e, *n*-butanol; f, ethanol; g, *n*-pentanol; h, nitrobenzene; i, petroleum ether; j, aniline; k, DMF; and l, chloroform solution); (**B**) Xerogels in DMF (a, TC18-Lu; b, TC16-Lu; and c, TC14-Lu).

It is well known that hydrogen bonding plays an important role in the formation of organogels [43-45]. At present, in order to further clarify this and investigate the effect of alkyl substituent chains on assembly, the spectra of xerogels of TC18-Lu were compared, as shown in Figure 8A. As for the spectrum of the TC18-Lu xerogel from petroleum ether, some main peaks were observed at 3,242, 2,918, 2,848, 1,709, 1,648, and 1,469 cm^−1^. These bands can be assigned to the N-H stretching, methylene stretching, carbonyl group stretching, amide I band, and methylene shearing, respectively [46-48], In comparison, in the spectrum of TC18-Lu in chloroform solution, the corresponding characteristic peaks appeared at 1,743 and 1,586 cm^−1^, respectively. The obvious shifts indicated the strong intermolecular hydrogen bonding interaction between imide compounds. In addition, the IR spectra of TC18-Lu, TC16-Lu, and TC14-Lu in DMF were compared, as shown in Figure 8B. One obvious change is the decrement of methylene stretching for TC16-Lu and TC14-Lu in comparison with TC18-Lu at 2,916 and 2,848 cm^−1^, which can be attributed to the number difference of alkyl substituent chains in molecular skeletons. It is interesting to note that the peak assigned to amide I band shifted to the positions of 1,658, 1,683, and 1,652 cm^−1^ for TC18-Lu, TC16-Lu, and TC14-Lu, respectively. The obvious changes indicated the formation of different H-bonds between imide groups in the gel state. This implied that there were differences in the strength of the intermolecular hydrogen bond interactions in these xerogels, even though they were from the same solvent system.

**Figure 9:**
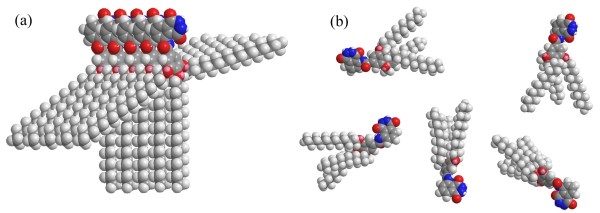
**Schematic pictures of assembly modes.** (**a**) TC18-Lu in organogels and (**b**) TC12-Lu in solution.

Considering the XRD results described above and the hydrogen bonding nature of the orderly aggregation of these imide compounds as confirmed by FT-IR, a possible assembly mode of TC18-Lu organogels was proposed and is schematically shown in Figure 9. As for the xerogel of TC18-Lu from different solvents, due to the strong intermolecular hydrophobic force between longer alkyl substituent chains and hydrogen bonding interaction between imides in the luminol part, this gelator can self-assemble to form orderly nanostructures. The calculated repeating unit with a length of about 2.0 nm was obtained. The obtained experimental value was in range of 2.0~2.1 nm, which was in good accordance with the calculation result. In addition, for the xerogels of TC14-Lu from DMF, with the decrement of alkyl substituent chains, the weaker intermolecular hydrophobic force between the alkyl chains of the neighboring molecules will not enable present gelators to orderly assemble as in the case of TC18-Lu and shows a shorter layer distance and more disorderly stacking unit. For the case of TC12-Lu, no gel can be prepared due to the shortest alkyl substituent chains, as shown in Figure 9b. Meanwhile, it should be noted that this phenomenon is similar to the results of recent reports [49,50]. Therein, the substituent groups in azobenzene residue or benzimidazole/benzothiazole imide derivatives can have a profound effect upon the gelation abilities and the as-formed nanostructures of the studied compounds. For the present system, the experimental results demonstrated again that the alkyl substituent chains had played a very important role in regulating the assembly modes and nanostructures in these organogels. Now the ECL properties generated by the present xerogels of these luminol derivatives in the presence of hydrogen peroxide are under investigation to display the relationship between the molecular structures, as-formed nanostructures, and ECL sensors.

## Conclusions

Some luminol imide derivatives with different alkyl substituent chains have been synthesized. Their gelation behaviors in various organic solvents can be regulated by changing the length and number of alkyl substituent chains. The experimental data demonstrated that the length of alkyl substituent chains linked to a benzene ring in these imide derivatives can have a profound effect upon the gelation abilities of these studied compounds. Longer alkyl chains in molecular skeletons in the present gelators are favorable for the gelation of organic solvents. Morphological studies revealed that the gelator molecules self-assemble into different aggregates from dot, flower, belt, rod, and lamella, to wrinkle with change of solvents. Spectral studies indicated that there existed different H-bond formations and hydrophobic force, depending on the alkyl substituent chains in molecular skeletons. The present research work affords a new useful exploration for the design and development of new versatile low molecular mass organogelators and soft matter for ECL biosensors with luminol functional groups.

## Competing interests

The authors declare that they have no competing interests.

## Authors’ contributions

TJ participated in the analysis and testing of the nanostructures. QH carried out the synthesis of compounds and characterization of organogels. QZ and FG supervised this work, helped in the analysis and interpretation of data, and, together with DX, worked on the drafting and revisions of the manuscript. TJ and QZ conceived the study and participated in its design and characterization. JZ participated in the design of the study and provided the analysis instruments. All authors read and approved the final manuscript.

## Authors’ information

TJ and QZ are associate professors. QH is an MD student. DX is a professor. FG is a professor and the dean of the School of Environmental and Chemical Engineering. JZ is a laboratory assistant in Yanshan University.
